# Eye/Head Tracking Technology to Improve HCI with iPad Applications

**DOI:** 10.3390/s150202244

**Published:** 2015-01-22

**Authors:** Asier Lopez-Basterretxea, Amaia Mendez-Zorrilla, Begoña Garcia-Zapirain

**Affiliations:** DeustoTech-Life Research Unit. DeustoTech Institute of Technology, University of Deusto, Avda. Universidades, 24. 48007 Bilbao, Spain; E-Mails: asilop_92@hotmail.com (A.L.-B.); mbgarciazapi@deusto.es (B.G.-Z.)

**Keywords:** HCI, eye/head tracking, blinking, iPad, Haar Cascade

## Abstract

In order to improve human computer interaction (HCI) for people with special needs, this paper presents an alternative form of interaction, which uses the iPad's front camera and eye/head tracking technology. With this functional nature/capability operating in the background, the user can control already developed or new applications for the iPad by moving their eyes and/or head. There are many techniques, which are currently used to detect facial features, such as eyes or even the face itself. Open source bookstores exist for such purpose, such as OpenCV, which enable very reliable and accurate detection algorithms to be applied, such as Haar Cascade using very high-level programming. All processing is undertaken in real time, and it is therefore important to pay close attention to the use of limited resources (processing capacity) of devices, such as the iPad. The system was validated in tests involving 22 users of different ages and characteristics (people with dark and light-colored eyes and with/without glasses). These tests are performed to assess user/device interaction and to ascertain whether it works properly. The system obtained an accuracy of between 60% and 100% in the three test exercises taken into consideration. The results showed that the Haar Cascade had a significant effect by detecting faces in 100% of cases, unlike eyes and the pupil where interference (light and shade) evidenced less effectiveness. In addition to ascertaining the effectiveness of the system via these exercises, the demo application has also helped to show that user constraints need not affect the enjoyment and use of a particular type of technology. In short, the results obtained are encouraging and these systems may continue to be developed if extended and updated in the future.

## 1. Introduction and Background

In recent years, the industries involved in the production, sale, use and servicing of smartphones and tablets have grown exponentially, with these smart devices becoming a feature of many people's everyday lives. However, the research and development behind much of this technology has not taken into account the interaction needs of certain user groups, such as people with disabilities and cerebral palsy.

The concept of Human Computer Interaction (HCI) refers to a discipline, which studies information exchange between people and computers by using software. HCI mainly focuses on design—assessing and implementing interactive technological devices that cover the largest possible number of uses [1].

The ultimate goal of HCI is to make this interaction as efficient as possible, looking to: minimize errors, increase satisfaction, lessen frustration, include users in development processes, work in multidisciplinary teams and run usability tests. In short, the goal is to make interaction between people and computers more productive.

New technologies have brought about a wave of health-related developments, and by using HCI, they meet the needs of different groups (people suffering from cerebral palsy, autism, Down syndrome and the elderly, *etc.*) [2]. Although these advances were unthinkable just a few years ago, they are gradually becoming a part of people's daily lives [3] and thanks to other concepts, such as ubiquitous computing, an attempt is being made to try and integrate IT into the individual's environment to the extent that all users may interact naturally with their devices—and extending forms of interaction beyond the classic ones, namely the mouse, keyboard, touch screen, synthesizers, voice recognition, *etc*. The touch screen is the device that is most used to interact with mobile devices, and this is not always easy as user psychomotor activity is clearly affected in disabled persons with class 3 and 4 functional capacity, although there are also major problems related to: sensitivity, cognition, communication, perception and behavioral disorders.

One of the technologies that can help to overcome the limitations of users with special needs (such as cerebral palsy) are all those that do not involve any physical action on the part of the user (hands or fingers). Other aspects such as the user's eyes and face provide data that can be interpreted by certain processing technologies.

All the data obtained is combined so as to give rise to a system that does not depend on the touch screen and is therefore adapted to the physical needs of some of the groups referred to above.

Eye tracking is currently being used in many fields, such as health and commercial studies. The process consists of measuring either the focus of attention (gaze) or eye movement in relation to the head. An eye tracker is a device for measuring the position of the eyes and eye movement [4]. The number of applications is infinite, some of which include [5]: a human computer interaction tool for the physically disabled, ergonomic studies, enhancement of sports performance, the clinical area (clinical diagnoses and correction of defects), leisure and videogames, and advertising and design studies.

Although technologies are in a state of continuous change, it seems that eye-tracking systems have still not undergone significant changes. At present, users can choose whether to employ a monitoring system by means of remote control, which implies a restriction of movements, or a fixed system mounted on the user's head (an uncomfortable and rather impractical system) [5,6].

The main problem with these eye-tracking systems is the limited range of (commercial) devices available on the market, which means that their prices are exorbitant, as shown in Table 1 (since eye-trackers depend on a PC or a device), and are therefore in many cases inaccessible for users in need.

Most of the devices shown in Table 1 are not only very expensive but are also common in research projects, although they are not very widely-used in commercial applications as the functions they offer go beyond interaction itself with the PC.

The study of eye movement is very widespread in different sectors and applications, as can be seen in Table 2.

As can be seen in Table 1, there has been an interest in eye tracking for some years now, with the first examples being in the 1990s, specifically 1996 and 1999. Subsequently, the use of eye tracking has been used mainly for usability and accessibility studies and, more recently, the latest ones in 2014 have already included the combination of eye tracker and information deriving from other devices [17].

Eye-tracking technology is therefore widely-applied, although hardly used in mobile devices, as can be seen in Table 3. Projects that make use of eye/head tracking in a given environment such as a tablet or smartphone are practically non-existent (2012–2014), and it is precisely the interaction with these devices that are socially widespread that has become a requirement for the previously-mentioned groups.

Evidently, there are difficulties that need to be identified and made known, such as the features of cameras and screens, although this is technically possible nowadays.

Thus, in this project, eye tracking is integrated into the system itself (iPad). A mobile computing system has been developed that makes use of mobile hardware and software. This system makes it possible to send data (image processing) via iPad without having to be connected to a fixed physical link.

With the aim of reducing the restrictions attached to the eye trackers mentioned above, open source bookstores and the tablet's front camera have been used, which cover the following points:

- They avoid depending on an external sensing system using the built-in camera.
- They minimize costs.
- They increase overall performance by integrating everything into the system.
- A special design is obtained for different groups of users.
- They have a tablet application.

## 2. Proposed Methods

This section contains a description of the materials used to develop the system, the tests run with users and the development methodology.

### 2.1. Components

The components used are described below. It mainly consists of the hardware and software that make up the system, with the users taking part in the tests and the questionnaires used for the tests.

#### (A) Hardware

The device used is the iPad tablet (Apple), more specifically the iPad 3. The portability, performance and design of the system itself were of the utmost importance in the choice of device. In addition, the experience gained by the authors in previous studies [19,20], in which convincing satisfactory results were obtained, has also been of great assistance.

Furthermore, being highly intuitive and interactive, the device is an extremely suitable tool for working on different skills with disabled users [20].

As for the sensing system—the camera—no external hardware was needed: the iPad's own integrated camera was able to be used. This is a front camera (Facetime HD), which, despite only having 2 Mpx, is sufficient for the processing involved in this project (assuming that the quality is not comparable to that of commercial systems). The iPad front camera is not designed to perform specific developments; therefore, Apple does not provide detailed information about the sensor, but OpenCV official webpage specifies that the camera is suitable for real time processing.

#### (B) Software

The iPad's base system is the IOS 7. Although the software also works in previous versions, it is specially designed for the IOS 7, taking advantage of the new possibilities offered with regard to resources and performance. The Xcode software development program was therefore used, with Objective C language.

On the other hand, the OpenCV open source bookstore was used for the ocular processing. This bookstore provides plentiful resources for both simple and advanced processing, with its performance being a significant advantage.

#### (C) Participants' Description

Twenty-two individuals in total took part in the tests. Twelve people had dark eyes and the remaining ten had light-colored eyes (blue or green). Age and gender were decisive factors when selecting participants, and the test was also conducted on eight people with glasses (of different colors), in order to ascertain the robustness of the system.

In this first phase, the tests to calculate the precision and reliability of the system have been conducted with not disabled users. In future tests, the authors have planned to try with disabled people, because ultimately they will be the main beneficiaries of the apps developed, including the proposed library.

### 2.2. Methods

#### 2.2.1. Defining the Venue

As regards the venue, tests were carried out in well-lit places (without direct lighting) so as to prevent any interference when processing. In terms of user position, they were all asked to be either seated or standing up, keeping the back straight, head up and looking straight ahead beside a window providing natural light.

In the image above an illustration of the position used in the tests can be seen, in the course of which the iPad was placed at a distance of between 20 and 30 cm from the user. By keeping the head up and looking straight ahead, any shadow was also avoided that might be caused by hair or eyebrows if the head were more tilted. The iPad was held in the hands during the tests (as the system does not need to be calibrated), although in an ideal situation it would be advisable to hold it using a support, thus preventing the user from becoming tired or making it impossible to use with their hands.

**Figure 1.**
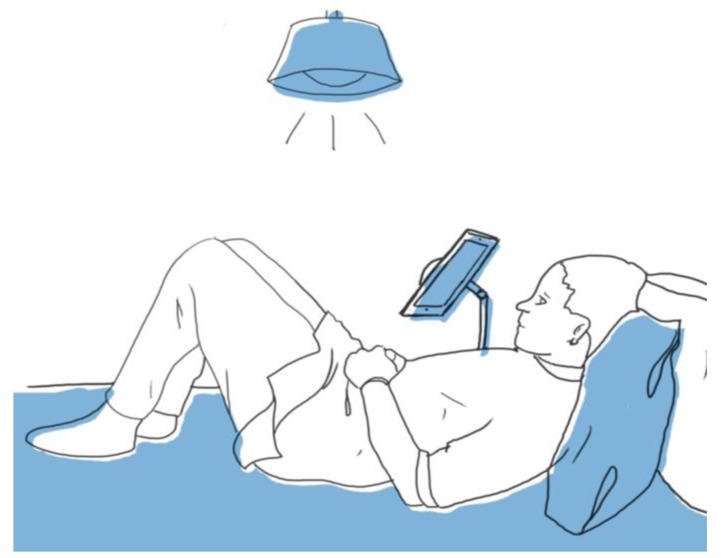
Tipped position with iPad stand.

**Figure 2.**
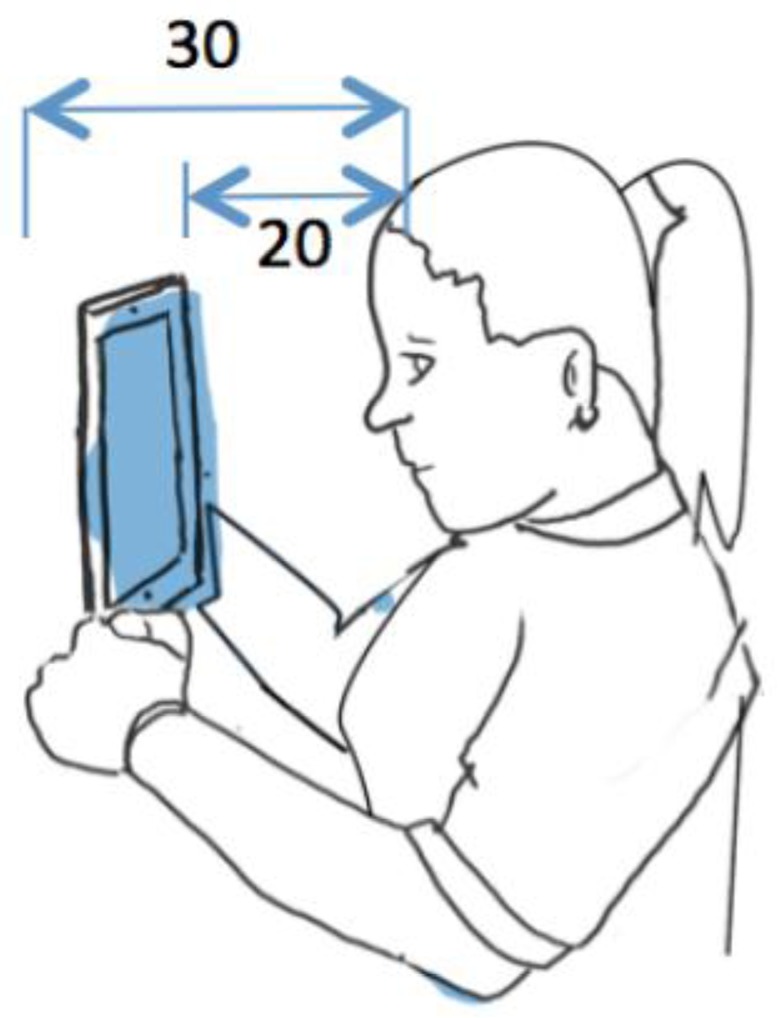
Suitable user-iPad position.

Figure 1 shows an example in which the user is lying down and the iPad has a support that ensures it remains in a fixed position. This position also enables there to be lighting that creates less shadow than in the case of the position described in Figure 2. The ideal distances between the iPad and the user remain the same as without support (between 20 and 30 cm).

The use of this support is suitable for use by users with some kind of disability who are unable to hold the iPad with their own hands, or who would not be able to guarantee the conditions described.

#### 2.2.2. Lighting Modes

The lighting systems used in the tests have always been artificial, preferably incandescent light. Owing to its features, fluorescent light suffers from shaking that increases the number of detection errors and harms the interaction.

#### 2.2.3. Test Methodology

Lastly, three exercises (described in Table 4) were created to carry out the tests with which the functioning of the different detection methods was able to be ascertained (and handling of the apps included in this option therefore validated), as follows.

## 3. Design

The design of the algorithm for the system is described in this section.

Certain situations were considered for this purpose [21]:

– Different lighting modes.
– Variable height and position of participants.
– Distance between the system and participants.

**Figure 3.**
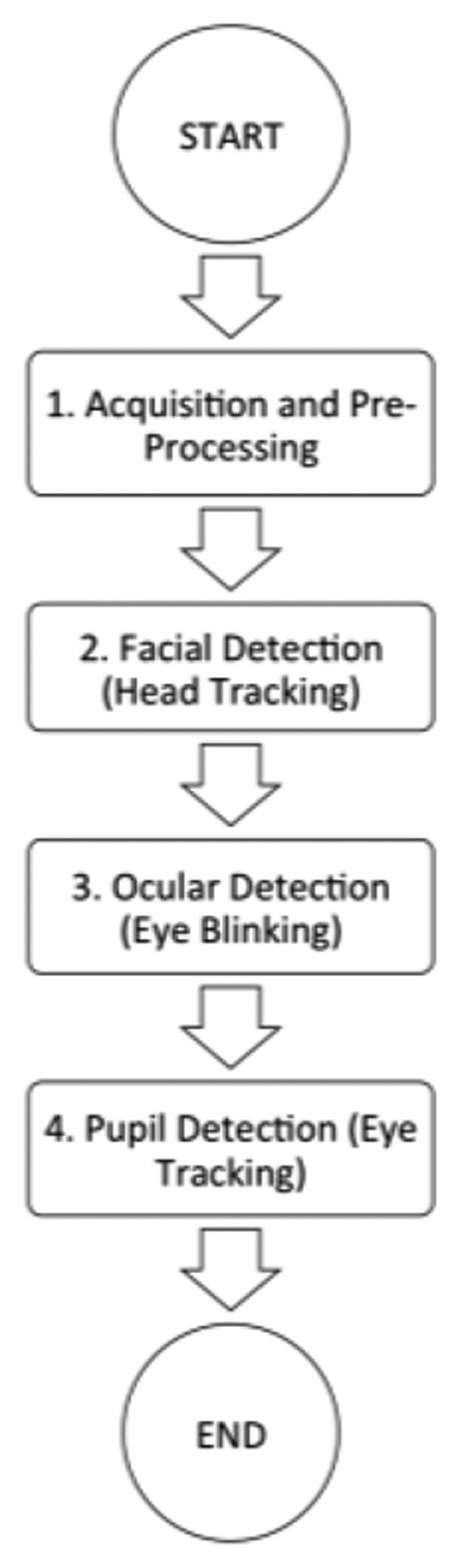
High-level block diagram.

The system design is divided into four major blocks, as shown in Figure 3.

The process described in Figure 3 was applied to each of the images deriving from the video source (the front camera of the iPad), thus making it a cyclical process. An open source bookstore (OpenCV) was developed to ensure that this system can be used in other applications that have already been created or are still to be created. In this project we developed a framework that makes use of current methodologies and proven techniques. The biggest challenge has been the effective incorporation of OpenCV and IOS frameworks. This library incorporates the processing of all the phases that are explained in more detail below.

### Stage 1: Acquisition and pre-processing

**Figure 4.**
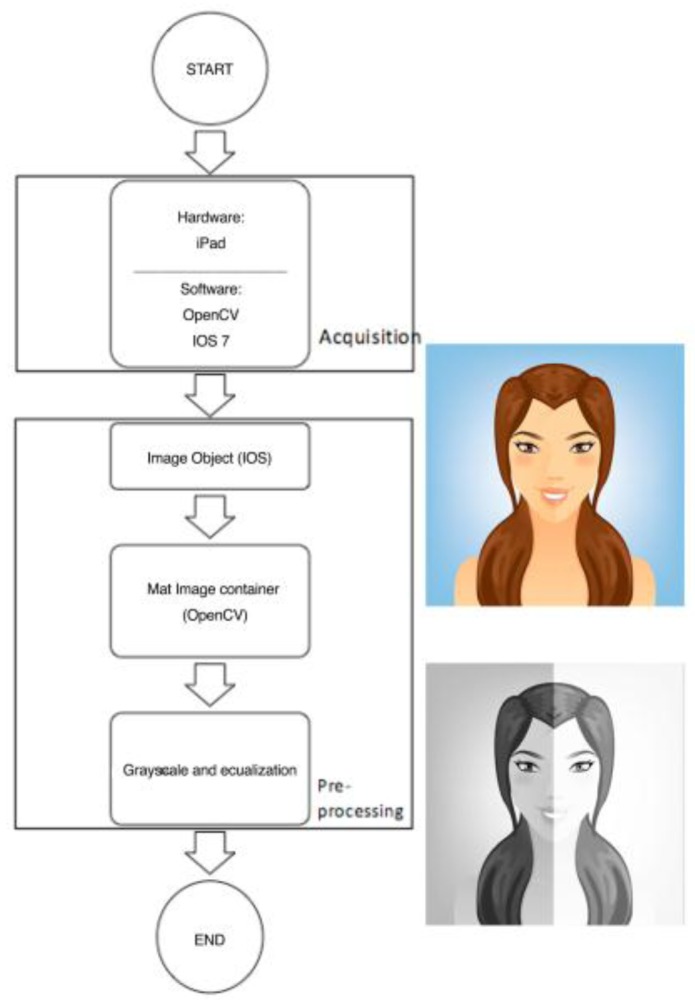
Low-level diagram of first stage.

The main purpose of this stage is to obtain different frames deriving from what the front camera of the iPad captures in real time on video. Subsequently, in the pre-processing stage, the image is passed onto a grayscale (reducing the number of channels from three to one) and is equalized in order to assist with detection. Figure 4 shows the diagram for the process in detail, together with a visual example of the progress made in the different stages.

### Stage 2: Face Detection

This is the stage when the processing of each of the images captured in Stage 1 gets underway. To do so, the Haar Cascade object detector [22] is used, which is specially trained to track faces. The Haar Cascade is a very effective method that was proposed by Paul Viola and Michael Jones in 2001 [21]. This is a machine-based learning process in which the cascade function has been trained from many positive images (images with faces) and negative (images without faces) images [23,24]. Once it has been trained, it is then used to detect objects in images.

The algorithm, which in the case of this project tracks the face and eyes [25–28], requires many positive and negative images in order to train the classifier.

**Figure 5.**
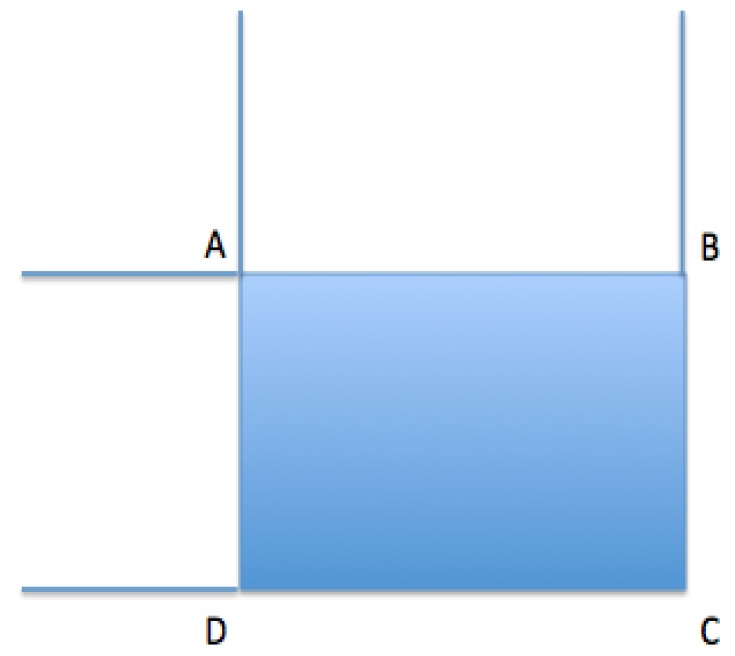
Haar Cascade integral images.

One of the greatest contributions made by Viola and Jones were the summed area tables or integral images (see Table 5). Integral images can be defined as two-dimensional search tables in the form of a matrix of the same size as the original image. Each element in the integral image contains the sum of all the pixels located in the upper left part of the original image (in relation to the element's position). This enables the sum of rectangular regions in the image to be calculated in any position or on any scale, using just four searches as it can be seen in Figure 5.

Thanks to this system, Haar characteristics of any image size can be used in constant time, thus reducing processing time and enhancing the system's performance. That is why this kind of template matching and classification techniques have proved effective in the field of eye tracking [27].

In this way, the different data attached to face tracking is provided by obtaining the image matrix, which will be analyzed in the following stage. Furthermore, data is also obtained at this point that enables head tracking. The following image shows Stage 2 in more detail.

**Figure 6.**
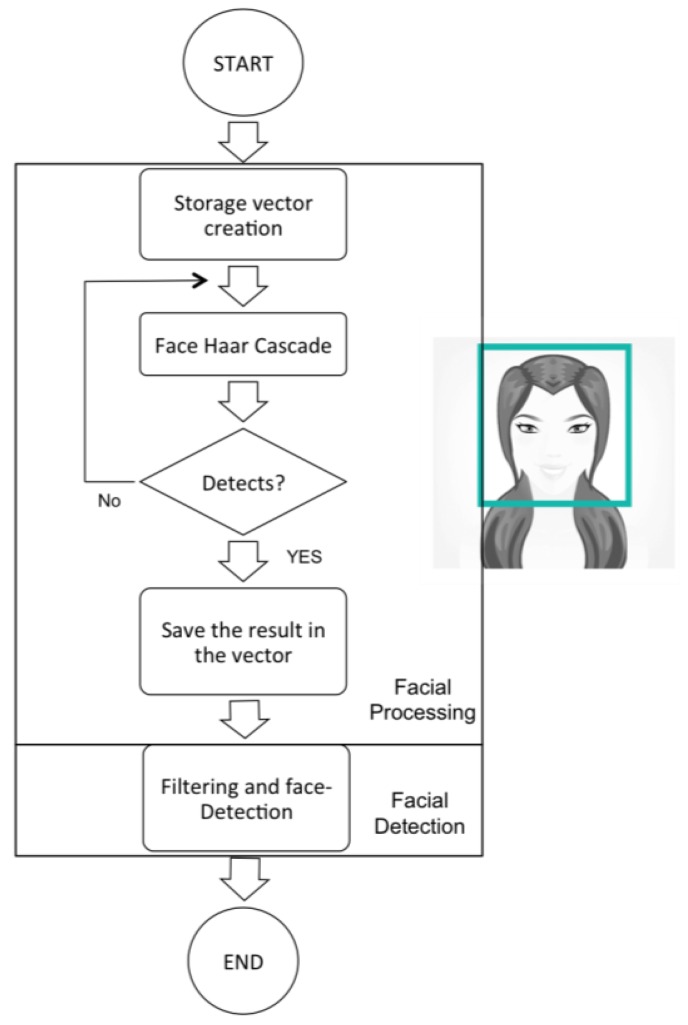
Low-level diagram of second stage.

Lastly, mention should be made of the algorithm created in this phase. This carries out the entire process described in Figure 6, together with the filtering.

#### headMovement Algorithm Description

**Figure 7.**
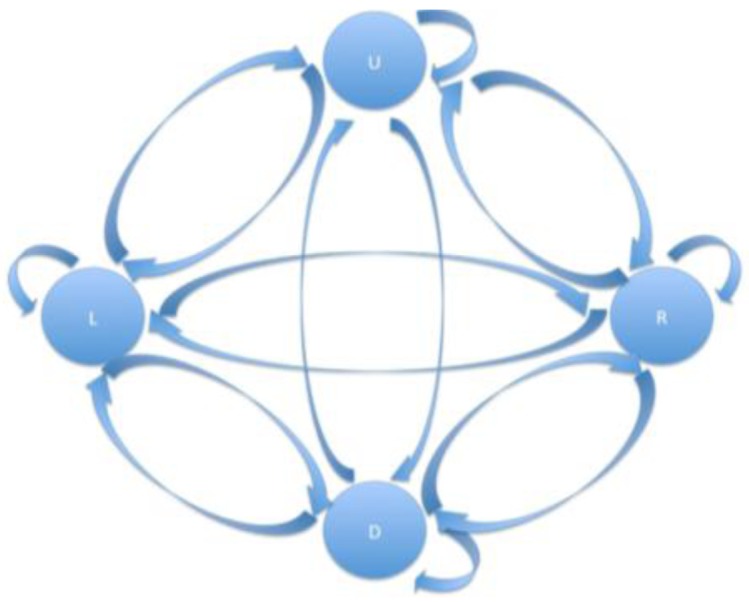
Flow chart for headMovement algorithm filtering.

This algorithm manages to return the position of the head to the screen. To this end, position x is analyzed and the vector detected and, using certain ranges (upper, lower and side limits), the position of the head is then determined. In this case, the algorithm detects 4 positions (up, down, left and right). A call to another method is included in order to filter all positions that arrive in real time, and this is applied to the following flow chart (see Figure 7).

Until the position is changed, the event does not take place, which in this case involves indicating its current position.

### Stage 3: Ocular Detection

In the third stage, we start from the matrix deriving from the face detection in such a way that the processing be reduced to the region of interest (ROI) of the head. The same OpenCV resource is once again used to detect both eyes, but in this case, a specially-designed Haar Cascade is used to detect them. A matrix with both eyes is obtained as a result of this.

**Figure 8.**
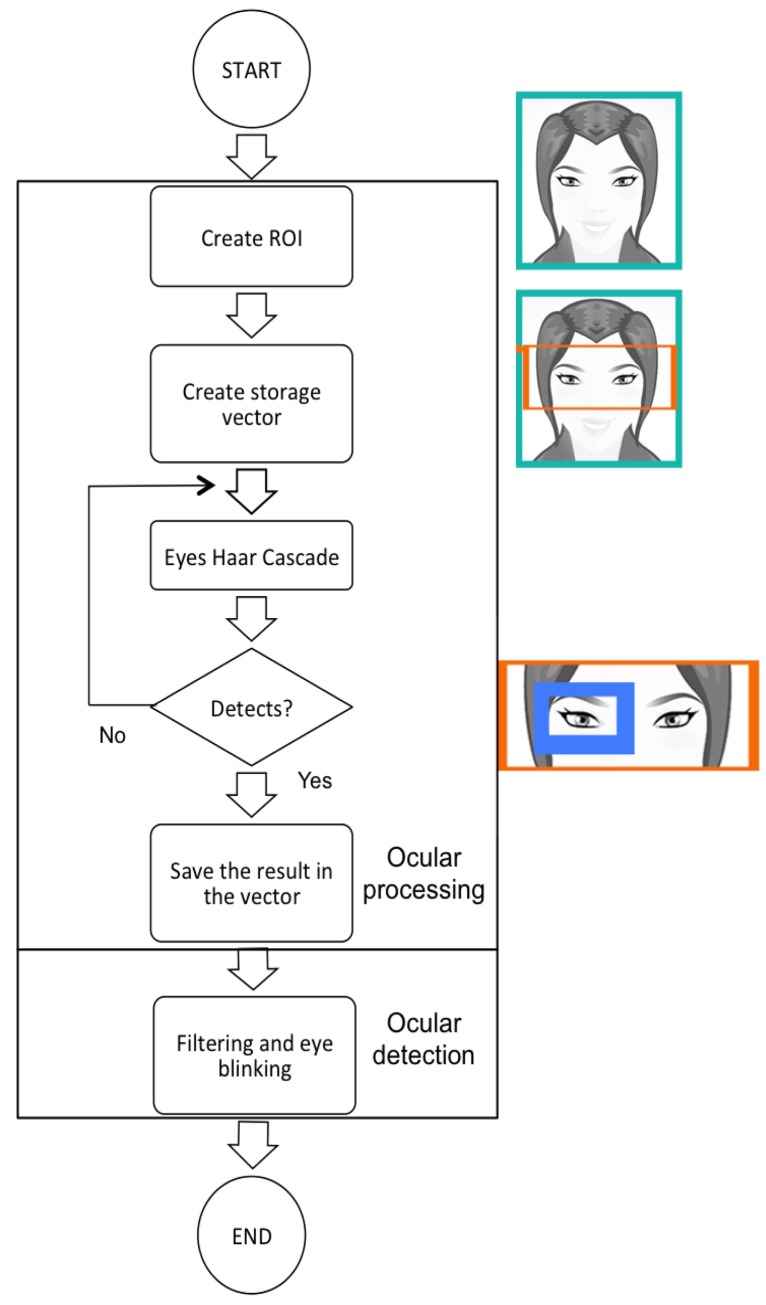
Low-level diagram of third stage.

A decision was made to work with just one eye so that the image matrix deriving from the Haar Cascade being applied is reduced to half its size, which means that processing time is also therefore reduced by half—critical in real-time applications. Lastly, this matrix is the one that passes on to the next stage. It is at this point where eye blinking is also obtained, to deduce whether the eye is open or shut. The process and end result are shown in detail in Figure 8.

The *eye detection* phase enables the algorithm that detects the eye blinking to be created. Below is described the *blinkControl* algorithm, which performs the phase 3 process together with its filtering stage:

#### blinkControl Algorithm Description

This algorithm is in charge of eye blinking:

- Eye open
- Eye shut
- Length of time that the eye is shut

**Figure 9.**
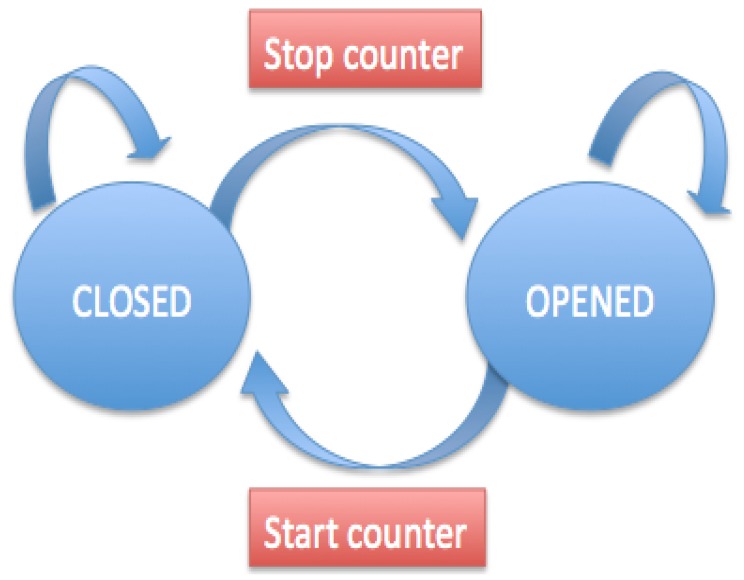
Flow chart for blinkControl algorithm filtering.

The call to a second method is also included that is in charge of filtering the different states. Figure 9 shows the flow chart that reflects how the filtering method works.

When the change from open to shut is detected, the meter starts to count and when it changes from shut to open it pauses, thus calculating the length of time that the eye is shut.

### Stage 4: Pupil Detection

**Figure 10.**
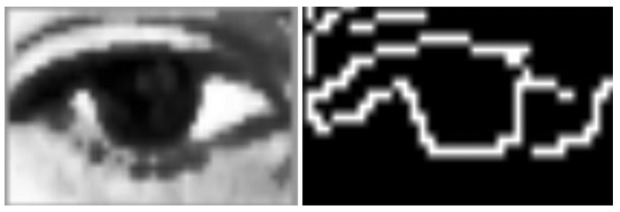
Eye region of interest.

Owing to the hardware requirements referred to in the first stage, different methodologies were checked in this stage [29–31], although some of them were not able to be applied owing to hardware limitations. This is the case with the Hough transform Circles, which is widely used to detect circles (pupil, as it can be seen in Figure 10, the image resolution makes it impossible to properly detect the pupil (circle).

As can be seen in Figure 10, the quality of the camera did not enable the Hough Circles transform to be suitably applied. The low resolution and existence of interference (eyelashes) after amplifying the image so much made it impossible to detect a circle.

Ultimately, it was decided to work with the matrix values from the previous phase, with the darkest eye (pupil) value being detected.

A system was developed to deduce the direction of gaze that avoids a previous calibration phase every time the system is used, as the latter is devised for the background with minimal user interference. To this end, the following technique was used, which only needs to be set up once by the user.

Following this stage, data is obtained for eye tracking purposes, ending with the three objectives that were set out at the beginning (head tracking, eye blinking and eye tracking).

**Figure 11.**
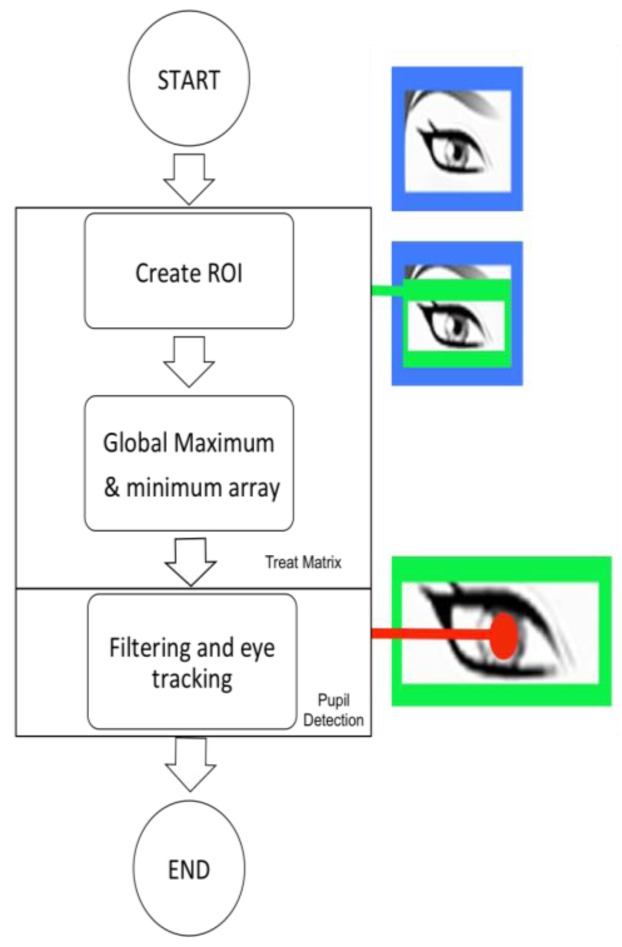
Low-level diagram of fourth stage.

Lastly, the process is repeated in order to detect the pupil, as shown in Figure 11.

To conclude this last phase of the fourth stage, an algorithm was once again developed that is in charge of eye tracking.

#### eyeControl Description

**Figure 12.**
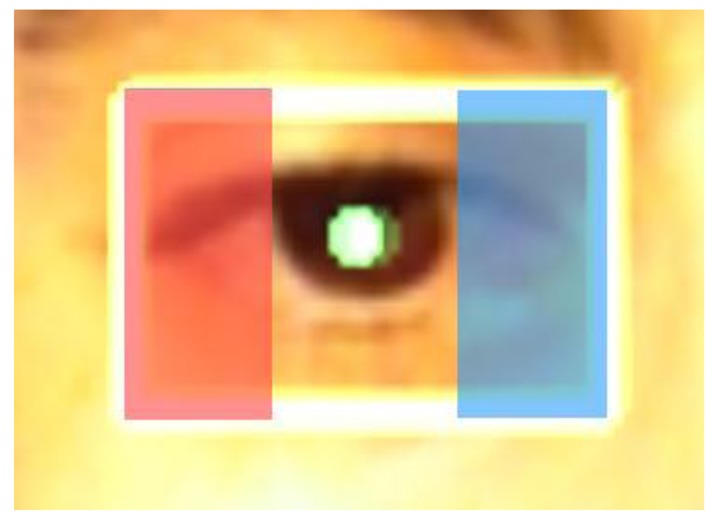
Eye tracking processing margins.

This algorithm is based on the pupil coordinate, and the width of the region of interest of the eye deduces the direction of gaze (left, center and right), as it can be seen in Figure 12.

Two margins were determined (they vary depending on the size of the user's eye). Once they have been adjusted, it is possible to detect whether the user is looking to the left, center or right if the central point of the pupil goes beyond any of the margins.

**Figure 13.**
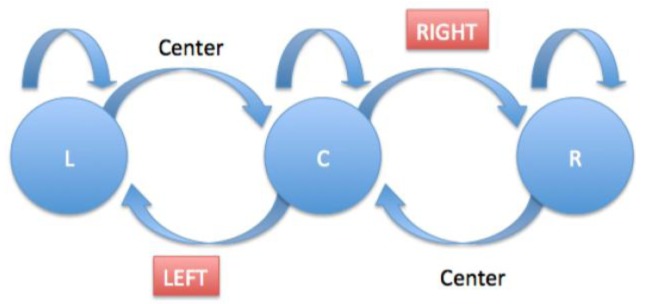
Flow chart for eyeControl algorithm filtering.

Additionally, the call to a second method is included that is in charge of filtering the different positions. Figure 13 shows the flow chart that reflects how the filtering method works.

Events occur when the change from center to left and from center to right are detected.

### 3.1. Lighting

From the different instances of detection, the most delicate is without doubt pupil detection. As has been explained in the previous section (Stage 4), given the hardware limitations (IR camera filter) and its quality, systems deemed more robust with lighting had to be disregarded (one of the major factors in real-time processing). Thus, certain ideal situations were used as a starting point in the design and development of this bookstore (described in Section 2.2 Methods). In such a scenario, the system works properly (see Section 4 Results), thus fulfilling the purpose of this study, although future work still needs to be done to improve it, involving paying close attention to the evolution of the hardware performance features of the devices.

#### 3.1.1. Demo Application

**Figure 14.**
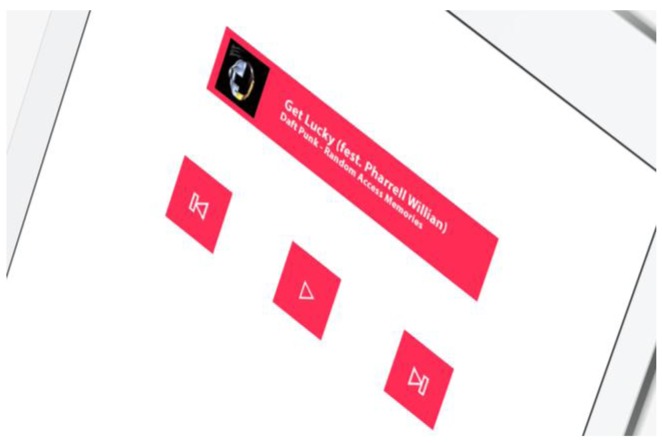
Demo music app.

A demo application (see Figure 14) was developed that enables the bookstore to be applied in a real test case.

The idea behind this application is to replicate the traditional iPad menu (music applications, images, books, the Internet, *etc.*) for all those groups of people who are unable to make use of a touch screen. We should recall that only applications for the system can be developed in the iPad, whereby it is not for instance possible to use the bookstore to control the native iPad music application. To this end, a separate application needs to be created that may work in the same way as the native one, albeit using the eye and face controls of the bookstore that has been created.

Only the music application was developed in the demo by way of an example. The following image shows the music application in more detail that was opened using three clear, simple controls.

- Play/Pause (opening and shutting the eye)
- Previous song (looking left)
- Next song (looking right)

#### 3.1.2. Demo Game Design

The application makes use of Apple native bookstores in order to gain access to songs stored on the iPad. If the user's left eye remains shut for more than a second (without blinking), the music starts to play randomly. If the user wishes to change song, they would look to the right so as to move on to the next song or to the left to play the previous one, and they are provided with informative data about the song they are currently listening to on the upper part. The library developed provides the results in real time, then, depending on the application you want to develop, those results could be displayed/used or not.

## 4. Results

In this section the technical results of the development of the application are explained in detail, as well as the objective results about users' performance in the exercises taken into consideration in the tests.

The descriptive statistics of the sample were analyzed using SPSS and, furthermore, inferential analyses were carried out using the Mann-Whitney statistical test. This test enabled the differences in results obtained from Exercises 2 and 3 (described in Section 2.2.3) to be analyzed according to eye color and use of glasses. In this case, Exercise 1 was not analyzed as it does not intervene directly in the eye in the face-tracking process.

The significance used was 0.05 (*p* = 0.05).

### 4.1. Descriptive Analysis

Owing to the small number of blue and green eyes in the samples, the dark brown and brown colors were grouped together in “Dark” and the green and blue ones in “Light-colored” (see Table 6).

In regards to the scores obtained from the exercises, there proved to be significant results. As can be seen in the following table in Exercise 2, a mean of 8.27 (see Table 7), from 0 to 10 was obtained (10 signifies that the 10 sequences in each exercise have been successfully carried out). In Exercise 3, the mean is lower, as more factors interfere in pupil detection than in ocular detection.

### 4.2. Inferential Analysis Results According to Eye Color

The differences in scores obtained from the exercises according to eye color are analyzed in this section. As can be observed in Table 8, significance is no less than 0.05, whereby there is no statistical evidence to suggest that there is any real difference between dark and light-colored eyes in terms of the scores obtained from the exercises. From the significance obtained from Exercise 3 it can be deduced that there are differences between both colors, but given the limited number of samples, this type of supposition cannot be assumed.

### 4.3. Inferential Analysis Results According to Use of Glasses

The differences in scores obtained from the exercises according to use of glasses are analyzed in this section, and they can be seen in Table 9.

As with Table 8, in this case significance is once again higher than the established limit (0.05), although in Exercise 3 significance is quite close albeit insufficient for the purpose of stating that there is no statistical evidence in the scores to really support any difference between using glasses or not.

Some of the images captured at random moments during the tests are shown below. Some special cases were also sought as erroneous detections.

**Figure 15.**
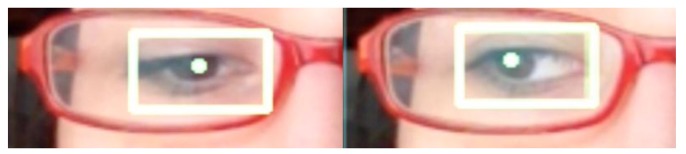
User with glasses 1.

**Figure 16.**
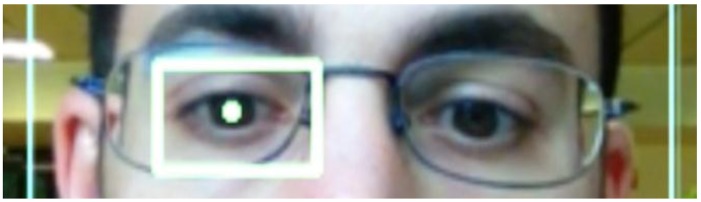
User with glasses 2.

Figures 15 and 16 show examples of the detection of two users with glasses.

**Figure 17.**
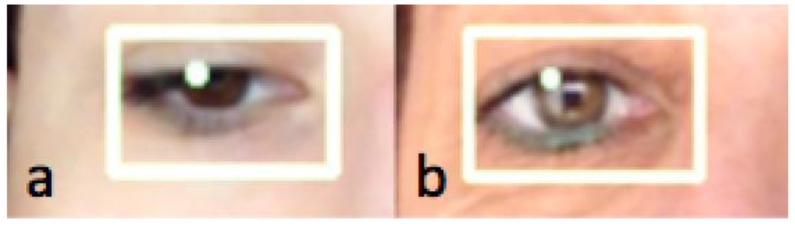
Erroneous detections.

Two cases were captured in the following cases, which show the extent of deviation of the detection:

In Figure 17, two cases can be seen that include the most common elements that may have a bearing on the end result:

- In the left image: eyelashes that may cover much of the eye and pupil or make it difficult to detect.
- In the right part of image (a): brightness in the eye caused by a more powerful, direct light.
- In the right part of image (b): made-up eyes (creation of dark areas that may interfere with the pupil).

Some of these positions provide erroneous data, although thanks to filtering of the fourth stage included in the bookstores, it proved possible to filter most of these erroneous detections. In any event, such deviations do not affect the system's performance (in Exercise 3, the mean deviation possibility with unfavorable results accounted for 9% of the exercises undertaken).

**Figure 18.**
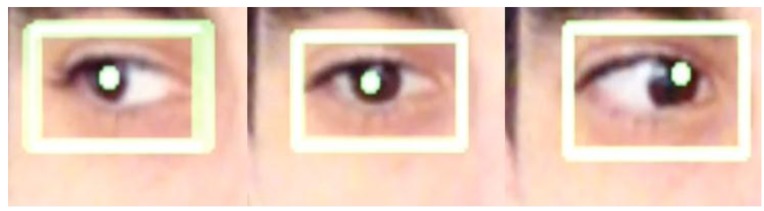
Gaze to the left, center and right.

Figure 18 above shows the example of another user; in this case with dark brown eyes and without glasses and with a distinction being clearly drawn between the three positions detected. It should be mentioned that in the case of the gaze to the left; the eye travels far less in that position than it does from center to right, as can be seen in the images. Thus, the one on the left needs to be more pronounced than the one on the right when selecting the margins that show at which part the user is looking.

## 5. Conclusions

In this section both the results obtained in the tests and the conclusions drawn obtained subsequently have been taken into account, so as to ultimately analyze future lines of research for this project.

Taking into consideration the results obtained in the tests and the exercises described in the previous point, the following conclusions have been drawn:

- Glasses constitute no hindrance, even when dark and colored ones were being used to try and cheat the system.
- Those eyes that were best detected were light-colored (green) ones. They obtained 90% accuracy in the most complex test (Exercise 3) and no erroneous detection was apparent.
- Face detection was 100% in all cases. Even under conditions of unsuitable light, the Haar Cascade method proved to be very effective [21].
- The results obtained from Exercise 3 depend on the accuracy of the eye detection that was worked on in Exercise 2. Thus, some of the errors from the third phase depend on proper detection of the eyes rather than of the pupil.
- Although all users were positioned at the same distance from the iPad (30 cm), the device's tilt and height of the iPad proved to be determining factors.

As far as general lines of research are concerned, the objectives set out in the project have been satisfactorily met. A bookstore was designed and implemented that enables there to be innovative and useful human–computer interaction at zero cost. An application was also created by way of a test with a view to applying the bookstore developed, with positive results being obtained.

This project is based on commercially-available hardware (iPad), which is why a specific suitable solution needed to be created for the resources available, taking into consideration both its advantages and disadvantages. Although the iPad at first glance meets all the requirements, the non-invasive eye-tracking system (infrared light) has not been able to be developed, as the front iPad camera contains an infrared filter, which makes it difficult to capture this type of light. As a result of this setback, processing was carried out directly on the image in color, with everything that entails.

The system is developed for IOS (the mobile operating system of iPhone and iPad), so it could also be used on the iPhone. Still, the quality of the front camera of the iPhone is of lower quality (1.2 Mpx) and the applications that are developed in the future are designed for the iPad (given their greater size). Anyway, the authors have also considered the option of testing and applications to the iPhone in the near future.

Lastly, the eye-tracking, eye-blinking and face-detection techniques were able to be applied, and the results expected were also obtained in the tests. However, certain lighting conditions are needed in order to properly apply some of these techniques (to prevent fake shadows), as stated in the Results section. A statistical survey was carried out with a view to show the system's accuracy regarding different eye colors and glasses, although the results proved to be insignificant.

Nonetheless, this project shows that technologies may be accessed by certain social groups if specially-designed products that have been devised for such purpose are created. It has also been possible to show that this project's limitations have been imposed by the hardware used rather than the software, which is an important point. Thus, it is hoped that manufacturers will increase the number of features and resources offered by their products as a result of this type of project, to the extent that there will be no barrier or limitation that might make it difficult to implement the systems.

Final remarks:

- When the original idea of this project was first considered, there was at the time no project that combined these new forms of interaction in a mobile terminal or tablet. At the present time, similar products are starting to emerge, which would seem to indicate that there is innovative technology out there that has a future.
- In the case of the demo that was developed, a decision was made to use the bookstore to control applications and browse them, thus replacing the need to use the touch screen, although these technologies can be expanded and applied in a wide range of areas (video games, entertainment, utility, assistance, *etc.*).
- The results obtained during development of the project and the tests carried out show that many factors play a part in most real-time image processing systems in the system's reliability. Having said this, a highly promising product has been obtained, and in this case the limiting factor has been the hardware.
- Open source resources have been used, which is why an attempt is made to share the resources created with the community by providing the relevant documentation.

## Figures and Tables

**Table 1.** Comparison of commercial eye trackers.

	**Tobii x2-60**	**Tobii X2-30**	**Tobiiglasses**	**The Eye Tribe**
	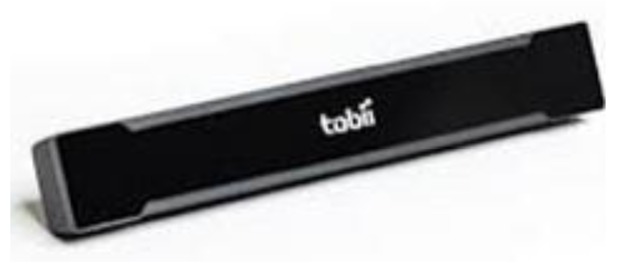	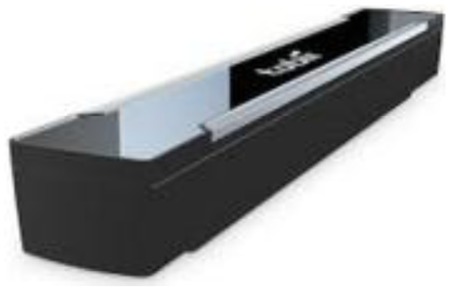	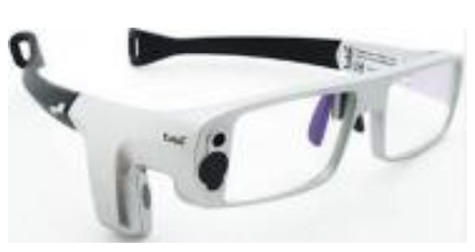	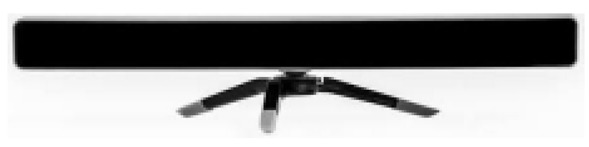
**Sample rate**	60 Hz	30 Hz	30	30 Hz y 60 Hz
**Latency**	<35 ms	50–70 ms	-	<20 ms a 60 Hz
**Recommended screen size**	Up to 25” (16:9)	Up to 25” (16:9)	-	Up to 24”
**Working distance**	40–90 cm	40–90 cm	60–250 cm	45 cm–75 cm
**Weight**	200 g	200 g	75 g	70 g
**Size**	184 × 28 × 23 mm	184 × 28 × 23 mm	123 × 83 × 32.5 mm	20 × 1.9 × 1.9 cm
**Software included**	Si	Si	Si	Si
**SDK**	Si	Si	Si	Si
**Price**	>40,000€	>20,000€	18.798€	75€
**Connection**	USB 2.0	USB 2.0	-	USB 3.0

**Table 2.** Tracking-related projects.

**Title**	**Authors**	**Year**	**Objectives**	**Devices/Sensor**
Effect of low alcohol concentration on visual attention span in street traffic [7]	Buser, A., Lachenmayr, B., Priemer, F., Langnau, A., Gilg, T.	1996	To demonstrate the effect of alcohol on drivers' concentration and attention span using an eye-tracking system	Eye tracker and IR light
openEyes. Low-cost head mounted eye-tracking solution [8]	Li, Dongheng, Babcock, Jason, Parkhurst, Dj	2006	Design and development of an open source eye-tracking system	Eye tracker
Research into eye-catching colours using eye tracking [9]	Mokryun Baik, Hyeon-Jeong Suk, Jeongmin Lee, Kyungah Choi	2013	Advertising and design studies	-
Using eye-tracking and support vector machine to measure learning attention span in eLearning [10]	Chien Hung Liu, Po Yin Chang, Chun Yuan Huang	2013	To detect the level of attention span in students' learning process in the absence of a supervisor	Eye tracker
Eye tracking in human-computer interaction and usability research: ready to deliver the promises [11]	Jacob, RJK, Karn, KS	2003	To use the eye-tracking technique as an interaction and usability tool with systems	Eye tracker
Real-time eye tracking and blink detection with USB cameras [12]	Chau, Michael, Betke, Margrit	2005	Use of eye tracking and eye blinking as a computer control system	Eye tracker
Hands-free interface to a virtual reality environment using head tracking [13]	Sing Bing Kang	1999	Use of head tracking for a hands-free browsing system in a computer-controlled environment	Camera and computer system
Driving with binocular visual field loss? A study of a supervised on-road parcours with simultaneous eye and head tracking [14]	Enkelejda Kasneci, Katrin Sippel, Kathrin Aehling, Martin Heister, Wolfgang Rosenstiel, Ulrich Schiefer, Elena Papageorgiou	2014	To assess the on-road driving performance of patients suffering from binocular visual field loss using a dual-brake vehicle, and to research into related compensatory mechanisms	-
A method to monitor eye and head tracking movements in college baseball players [15]	Fogt, Nicklaus F.; Zimmerman, Aaron B.	2014	To develop a method to measure horizontal gaze tracking errors (based on synchronized eye and head tracking recordings) as subjects viewed many pitched balls, and to assess horizontal eye, head, and gaze tracking strategies of a group of Division 1 college baseball players	Video eye tracker and an inertial sensor
Head pose estimation using a coplanar face model for human computer interaction [16]	Jin-Bum Kim, Hong-In Kim, Rae-Hong Park	2014	To create an algorithm to estimate the head pose without *a priori* having any information about the specific user, such as geometrical information about the face. This algorithm can be used in HCI applications for the general user	-

**Table 3.** Mobile Eye-tracking projects.

**Company/Product**	**Mobile Operative System**	**Date Application Launched**	**Objectives**	**Devices/Sensor**	**SDK**	**Camera**
Startup Umoove [17]	IOS and Android	13 February 2014	Natural interaction with face and eyes for mobile applications, business associations and revolutionary analytical platforms	Eye tracking and head tracking	Not included	Internal (front)
Fixational [18]	IOS and Android	3 September 2012: application for capturing images: the reading application has not yet been launched	To capture images via eye blinking E-book reader controlled by eye tracking	Eye tracking	Included	Internal (front)

**Table 4.** Test exercises.

**Exercise**	**Objective**	**Description**
Exercise 1	Working on face detection (face tracking)	Exercise comprising a sequence of ten movements (moving the head up, down, left and right)
Exercise 2	Working on ocular detection (eye blinking)	Exercise comprising ten sequences involving opening and shutting of eyes with different margins of time
Exercise 3	Working on pupil detection (eye tracking)	Exercise comprising a sequence of ten visualizations (looking up, down, left and right)

**Table 5.** Integral images.

Sum = I (C) + I (A) − I(B) − I(D).
//A, B, C and D refer to the points in the following image:

**Table 6.** Description of the sample (*n* = 22).

**Variables**		**Frequency (%)**
Eye color	Dark	54.50
	*Light-colored*	*45.50*

Glasses	No	63.64
	*Yes*	*36.36*

**Table 7.** Description of scores obtained from Exercises 2 and 3 (*n* = 22).

**Variable**	**Mean**	**Max**	**Min**
Score Exercise 2	8.27	10.00	6.00
Score Exercise 3	7.45	9.00	5.00

**Table 8.** Differences in mean scores according to eye color.

	**Eye Color**	**N**	**Mean**	**U of Mann–Whitney**	**P (Significance)**
Score Exercise 2	Dark	12	8.33	54.00	0.722
	Light-colored	10	8.20		

Score Exercise 3	Dark	12	7.25	46.50	0.381
	Light-colored	10	7.70		

**Table 9.** Differences in mean scores according to use of glasses.

	**Glasses**	**N**	**Mean**	**U of Mann–Whitney**	**P (Significance)**
Score Exercise 2	Yes	8	7.87	36.50	0.188
	No	14	8.50		

Score Exercise 3	Yes	8	6.87	30.00	0.082
	No	14	7.78		
